# Predictors of mild cognitive impairment in older adults living with HIV and multimorbidity: a comparative analysis using logistic regression and decision tree models

**DOI:** 10.3389/fpubh.2026.1837495

**Published:** 2026-05-25

**Authors:** Chunxing Ge, Huiying Gao, Lanting Xia, Yawen Wang, Meiyin Zou, Xiangyun Qian

**Affiliations:** 1 School of Nursing and Rehabilitation, Nantong University, Nantong, China; 2 Affiliated Nantong Hospital 3 of Nantong University (Nantong Third People’s Hospital), Nantong, China

**Keywords:** cognitive impairment, decision tree, HIV/AIDS, logistic regression, multimorbidity, older adults

## Abstract

**Background:**

With the aging of the global population of people living with HIV (PLWH), cognitive impairment has emerged as an important public health concern. Older adults with HIV frequently experience multimorbidity, which may further increase the risk of mild cognitive impairment (MCI). However, studies exploring predictors of MCI among older adults living with HIV and multimorbidity remain limited.

**Objective:**

To investigate the prevalence and factors associated with screening-defined mild cognitive impairment (MCI) among older adults living with HIV and multimorbidity, and to explore the potential predictive value of logistic regression and decision tree models.

**Methods:**

A cross-sectional study was conducted among 327 older adults (aged ≥50 years) living with HIV and at least one comorbid chronic condition. Sociodemographic characteristics, clinical information, self-management ability, and social support were collected through structured questionnaires. Cognitive function was assessed using the Montreal Cognitive Assessment (MoCA), and dementia was excluded using the Mini-Mental State Examination (MMSE). Univariate analysis and multivariable logistic regression were performed to identify factors associated with MCI. A CHAID decision tree model with 10-fold cross-validation was constructed to explore hierarchical relationships among predictors. Receiver operating characteristic (ROC) curves were used to assess model performance.

**Results:**

Among the 327 participants, the prevalence of screening-defined MCI was 48.9%. Multivariable logistic regression analysis showed that older age, female sex, hypertension, monthly income of 3,001–5,999 RMB, and a higher number of comorbidities were significantly associated with an increased risk of cognitive impairment (*p* < 0.05). In contrast, higher education level, HIV knowledge learning experience, greater social support, and better daily living management ability were protective factors (*p* < 0.05). The decision tree model identified five key predictors, including number of comorbidities, education level, age, hypertension, and HIV knowledge learning experience, with number of comorbidities being the most important splitting variable. The area under the ROC curve (AUC) of the logistic regression model was 0.961, which was significantly higher than that of the decision tree model (0.916; *p* < 0.01).

**Conclusion:**

Screening-defined cognitive impairment is highly prevalent among older adults living with HIV and multimorbidity. Factors including the number of comorbidities, age, education level, HIV knowledge learning experience, and hypertension were identified as important correlates of cognitive impairment, with consistent findings across logistic regression and decision tree analyses. Both models demonstrated acceptable discriminatory ability within the study sample; however, these findings should be interpreted as exploratory given the lack of external validation. Overall, the results may contribute to the early identification of individuals at higher risk of cognitive impairment and provide a basis for developing targeted interventions. Further studies with rigorous validation are warranted to confirm the generalizability of these findings.

## 1 Introduction

Population aging has become a major global demographic trend (1). In China, the number of individuals aged 60 years and older has exceeded 300 million, accounting for approximately 22% of the total population (2). Aging is often accompanied by an increased prevalence of chronic diseases, and more than one-third of older adults are affected by two or more chronic conditions (3). The coexistence of multiple chronic diseases, commonly referred to as multimorbidity, has become an increasingly important challenge for healthcare systems (4).

In recent years, the age distribution of people living with HIV (PLWH) has shifted significantly due to improvements in antiretroviral therapy (ART), which have substantially prolonged life expectancy. International studies typically define individuals aged 50 years and older as older adults living with HIV (5). In China, surveillance data indicate that the proportion of newly reported HIV infections among individuals aged ≥50 years increased from 22% in 2011 to 48.1% in 2022 (6). As the population of older adults living with HIV continues to grow, multimorbidity has become increasingly prevalent in this group.

Cognitive impairment is one of the most common neurological complications associated with HIV infection. In people living with HIV, cognitive impairment is commonly conceptualized within the framework of HIV-associated neurocognitive disorders (HAND), which includes asymptomatic neurocognitive impairment (ANI), mild neurocognitive disorder (MND), and HIV-associated dementia (HAD) (7). This framework incorporates both neuropsychological performance and functional impairment and is widely used in HIV-related cognitive research.

Older adults living with HIV are particularly vulnerable to cognitive decline due to the combined effects of aging, HIV-related neurotoxicity, and chronic comorbid conditions. While the HAND framework provides an important disease-specific classification, the concept of mild cognitive impairment (MCI) is widely used in aging research to capture early cognitive decline prior to dementia.

Previous studies have shown that chronic diseases such as hypertension, diabetes, and cardiovascular diseases may contribute to cognitive impairment through mechanisms including vascular injury, chronic inflammation, and metabolic dysregulation (8). Conversely, cognitive impairment may negatively affect medication adherence, self-management ability, and disease control, thereby further worsening health outcomes (9). Despite the growing concern regarding cognitive impairment in older adults with HIV, most existing studies have focused on single disease factors rather than the combined effects of HIV and multimorbidity. Moreover, relatively few studies have developed predictive models to identify individuals at high risk of cognitive impairment in this population.

Machine learning approaches, such as decision tree models, provide intuitive and interpretable tools for identifying hierarchical relationships among risk factors, while traditional statistical models such as logistic regression remain widely used for risk prediction. Therefore, this study aimed to investigate the prevalence of screening-defined cognitive impairment among older adults living with HIV and multimorbidity. Logistic regression and CHAID decision tree models were applied to explore these factors and to provide preliminary insights into their hierarchical relationships. The findings of this study may contribute to the early identification of high-risk individuals and provide evidence for the development of targeted screening and intervention strategies for cognitive impairment among older adults living with HIV.

## 2 Methods

### 2.1 Study design and participants

This cross-sectional study was conducted at an infectious disease outpatient clinic in Jiangsu Province, China, between October and December 2025. Participants were recruited using a convenience sampling method. The inclusion criteria were as follows: (1) screened using the Montreal Cognitive Assessment (MoCA) (10) with a score <25; (2) aged ≥50 years and met the diagnostic criteria of the Chinese Guidelines for the Diagnosis and Treatment of HIV/AIDS (2024 edition) (11); (3) having at least one chronic disease in addition to HIV infection; (4) able to communicateand complete the questionnaire independently or with assistance; (5) willing to participate and provided informed consent. The exclusion criteria included: (1) confirmed diagnosis of dementia; (2) history of severe brain injury, brain tumor or major psychiatric disorders; (3) substance abuse; (4) participation in similar studies previously. This study was approved by the Ethics Committee of Nantong Third People’s Hospital (Approval No. EK2025098). Written informed consent was obtained from all participants prior to data collection.

### 2.2 Sample size estimation

The sample size was estimated using Kendall’s method (12) for multivariate analysis, which recommends that the sample size should be 5–10 times the number of candidate variables. In this study, a total of 43 candidate variables were considered; therefore, the minimum required sample size was estimated to be between 215 and 430 participants. To account for a potential 20% rate of invalid or incomplete responses, the target sample size was further increased. Finally, 346 participants were recruited, and 327 valid cases were included in the final analysis, which met the minimum sample size requirement for multivariate analysis. In addition, the number of outcome events was sufficient to support multivariable logistic regression analysis.

### 2.3 Data collection

Data were collected through face-to-face interviews conducted by trained investigators. Participants completed questionnaires on sociodemographic characteristics, clinical information, self-management ability, and social support. For participants who were unable to complete the questionnaire independently, investigators read the questions aloud and recorded the responses. Cognitive function assessment was conducted separately by trained evaluators in a quiet and comfortable environment to ensure accuracy.

### 2.4 Measurements

#### 2.4.1 Sociodemographic and clinical characteristics

A structured questionnaire was used to collect information including age, sex, residence, education level, marital status, employment status, monthly income, infection route, and payment method for medical expenses. Clinical variables included duration of HIV diagnosis, antiretroviral therapy (ART) duration, CD4^+^ T-cell count, viral load, and comorbid conditions such as hypertension, diabetes, hyperlipidemia, and chronic kidney disease. The number of comorbidities was calculated for each participant.

#### 2.4.2 Self-management ability

Self-management ability was assessed using the HIV/AIDS Self-Management Scale, developed by Wu Chunyan (13). The scale consists of 30 items across five dimensions: daily life management, disease knowledge management, symptom management, treatment adherence management, emotional cognitive management. Each item is rated on a 5-point Likert scale ranging from 1 (“never”) to 5 (“always”). Higher scores indicate better self-management ability. The scale has demonstrated good reliability and validity, with a Cronbach’s *α* coefficient of 0.889.

#### 2.4.3 Social support

Social support was measured using the Social Support Rating Scale (SSRS) developed by Xiao Shuiyuan (14). The scale includes 10 items and assesses three dimensions: objective support, subjective support and utilization of support. The total score ranges from 12 to 66. Social support levels were categorized as: low (≤22); moderate (23–44); high (≥45). Higher scores indicate greater perceived social support.

#### 2.4.4 Cognitive function assessment

Cognitive function was assessed using two widely used screening tools: Montreal Cognitive Assessment (MoCA) and Mini-Mental State Examination (MMSE). The MoCA is considered more sensitive for detecting mild cognitive impairment (MCI) (15), whereas the MMSE has higher specificity for dementia screening (16).

In this study, the MMSE was used to exclude participants with dementia based on education-adjusted cutoff scores: ≤17 for illiterate individuals, ≤20 for those with primary school education, and ≤24 for those with secondary school education or above (17). Participants with MoCA scores < 25 were classified as having screening-defined MCI (18). As cognitive function was assessed using screening instruments alone, the identified MCI should be interpreted as screening-defined cognitive impairment rather than a formal clinical diagnosis.

### 2.5 Statistical analysis

All statistical analyses were performed using SPSS version 27.0. A two-sided significance level of *p* < 0.05 was considered statistically significant. Categorical variables were presented as frequencies and percentages and compared using the chi-square test or Fisher’s exact test. Continuous variables that were not normally distributed were presented as median and interquartile range (IQR) and compared using the Mann–Whitney U test.

Univariate analysis was performed to identify variables significantly associated with MCI. Variables with *p* < 0.05 in the univariate analysis were included in the multivariable logistic regression model to identify independent predictors of MCI. Odds ratios (ORs) and 95% confidence intervals (CIs) were calculated for each independent variable.

A CHAID decision tree model was constructed to explore hierarchical relationships among predictors of MCI. The maximum tree depth was set to six levels, with a minimum parent node size of 44 and a minimum child node size of 22 to ensure adequate sample sizes within nodes. Ten-fold cross-validation was applied to evaluate the predictive performance of the decision tree model (19).

To compare the predictive performance of the logistic regression and decision tree models, receiver operating characteristic (ROC) curves were generated, and the area under the curve (AUC) was calculated. Differences between the AUCs of the two models were assessed using the DeLong test.

## 3 Results

### 3.1 Participant characteristics

A total of 346 questionnaires were distributed in this study. According to the inclusion and exclusion criteria, 9 invalid questionnaires were excluded. In addition, 10 questionnaires were removed due to missing or incorrectly completed responses. Finally, 327 valid questionnaires were included in the analysis, yielding an effective response rate of 94.5%.

Among the participants, individuals aged 60–69 years accounted for 46.8% (153/327), males accounted for 78.0% (255/327), and 75.2% (246/327) resided in rural areas. The prevalence of screening-defined MCI was 48.9% (160/327). Regarding multimorbidity, 58.4% (191/327) of participants had one comorbid condition, while 35.8% (117/327) had two comorbid conditions. Detailed characteristics of the participants are presented in Table 1.

**Table 1:** Univariate analysis of factors associated with mild cognitive impairment among older adults living with HIV/AIDS and multimorbidity.

Variable		Total, n (%)	NC (*n* = 167)	MCI (*n* = 160)	Test statistic	*p* value
Age(years)	50–59	128 (39.1)	87 (68.0%)	41 (32.0%)	χ^2^ = 27.449	<0.001^*^
	60–69	153 (46.8)	67 (43.8%)	86 (56.2%)		
	≥70	46 (14.1)	13 (28.3%)	33 (71.7%)		
Sex	Male	255 (78.0)	143 (56.1%)	112 (43.9%)	χ^2^ = 11.624	<0.001^*^
	Female	72 (22.0)	24 (33.3%)	48 (66.7%)		
BMI	Underweight	16 (4.9)	7 (43.8%)	9 (56.3%)	χ^2^ = 1.015	0.798
	Normal weight	170 (52.0)	85 (50.0%)	85 (50.0%)		
	Overweight	114 (34.9)	62 (54.4%)	52 (45.6%)		
	Obesity	27 (8.3)	13 (48.1%)	14 (51.9%)		
Residence	Urban	81 (24.8)	52 (64.2%)	29 (35.8%)	χ^2^ = 7.425	0.006^*^
	Rural	246 (75.2)	115 (46.7%)	131 (53.3%)		
Education level	Primary school or below	75 (22.9)	9 (12.0%)	66 (88.0%)	χ^2^ = 84.994	<0.001^*^
	Junior high school	157 (48.0)	79 (50.3%)	78 (49.7%)		
	High school and above	95 (29.1)	79 (83.2%)	16 (16.8%)		
Marital status	Single	2 (0.6)	1 (50.0%)	1 (50.0%)	-	0.089^a^
	Married	266 (81.3)	139 (52.3%)	127 (47.7%)		
	Divorced	28 (8.6)	17 (60.7%)	11 (39.3%)		
	Widowed	31 (9.5)	10 (32.3%)	21 (67.7%)		
Employment status in the past 3 months	Unemployed	167 (51.1)	62 (37.1%)	105 (62.9%)	χ^2^ = 26.953	<0.001^*^
	Occasional or short-term employment	15 (4.6)	11 (73.3%)	4 (26.7%)		
	Stable long-term employment	145 (44.3)	94 (64.8%)	51 (35.2%)		
Living arrangement	Living alone	63 (19.3)	33 (52.4%)	30 (47.6%)	χ^2^ = 0.054	0.817
	Not living alone	264 (80.7)	134 (50.8%)	130 (49.2%)		
Monthly income (RMB)	≤3,000	125 (38.2)	41 (32.8%)	84 (67.2%)	χ^2^ = 39.713	<0.001^*^
	3,001–5,999	153 (46.8)	85 (55.6%)	68 (44.4%)		
	6,000–10,000	39 (11.9)	34 (87.2%)	5 (12.8%)		
	>10,000	10 (3.1)	7 (70.0%)	3 (30.0%)		
Health insurance type	Self-paid	9 (2.8)	6 (66.7%)	3 (33.3%)	-	<0.001^a*^
	Urban resident basic medical insurance	158 (48.3)	98 (62.0%)	60 (38.0%)		
	New rural cooperative medical scheme	160 (48.9)	63 (39.4%)	97 (60.6%)		
Smoking	Yes	57 (17.4)	35 (61.4%)	22 (38.6%)	χ^2^ = 2.950	0.086
	No	270 (82.6)	132 (48.9%)	138 (51.1%)		
Alcohol consumption	Yes	56 (17.1)	27 (48.2%)	29 (51.8%)	χ^2^ = 0.221	0.639
	No	271 (82.9)	140 (51.7%)	131 (48.3%)		
Duration since HIV diagnosis	<1 year	11 (3.4)	7 (63.6%)	4 (36.4%)	χ^2^ = 1.797	0.773
	1–5 years	160 (48.9)	78 (48.8%)	82 (51.2%)		
	>5–10 years	114 (34.9)	61 (53.5%)	53 (46.5%)		
	>10–15 years	30 (9.2)	14 (46.7%)	16 (53.3%)		
	>15 years	12 (3.7)	7 (58.3%)	5 (41.7%)		
HIV transmission route	Men who have sex with men (MSM)	122 (37.3)	76 (62.3%)	46 (37.7%)	-	0.001^a^*
	Heterosexual transmission	190 (58.1)	88 (46.3%)	103 (53.7%)		
	Other routes	15 (4.6)	3 (20.0%)	12 (80.0%)		
Duration of antiretroviral therapy (ART)	≤6 months	13 (4.0)	7 (53.8%)	6 (46.2%)	χ^2^ = 2.451	0.484
	7–12 months	25 (7.6)	10 (40.0%)	15 (60.0%)		
	13–18 months	11 (3.4)	4 (36.4%)	7 (63.2%)		
	>18 months	278 (85.0)	146 (52.5%)	133 (47.5%)		
CD4 + T-cell count (cells/μL)	≤200	29 (8.9)	13 (44.8%)	16 (55.2%)	χ^2^ = 2.065	0.559
	201–349	49 (15.0)	25 (51.0%)	24 (49.0%)		
	350–499	102 (31.2)	48 (47.1%)	54 (52.9%)		
	≥500	147 (45.0)	81 (55.1%)	66 (44.9%)		
HIV viral load	Detectable	39 (11.9)	17 (43.6%)	22 (56.4%)	χ^2^ = 0.992	0.319
	Undetectable	288 (88.1)	150 (52.1%)	138 (47.9%)		
Diabetes mellitus	Yes	104 (31.8)	32 (30.8%)	72 (69.2%)	χ^2^ = 25.152	<0.001^*^
	No	223 (68.2)	135 (60.5%)	88 (39.5%)		
Hypertension	Yes	129 (39.4)	33 (25.6%)	96 (74.4%)	χ^2^ = 55.390	<0.001^*^
	No	198 (60.6)	134 (67.7%)	64 (32.3%)		
Coronary heart disease	Yes	21 (6.4)	11 (52.4%)	10 (47.6%)	χ^2^ = 0.015	0.901
	No	306 (93.6)	156 (51.0%)	150 (49.0%)		
Hepatitis C	Yes	8 (2.4)	7 (87.5%)	1 (12.5%)	-	0.067^a^
	No	319 (97.6)	160 (50.2%)	159 (49.8%)		
Hepatitis B	Yes	15 (4.6)	9 (60.0%)	6 (40.0%)	χ^2^ = 0.502	0.479
	No	312 (95.4)	158 (50.6%)	154 (49.4%)		
Anxiety	Yes	15 (4.6)	11 (73.3%)	4 (26.7%)	χ^2^ = 3.118	0.077
	No	312 (95.4)	156 (50.0%)	156 (50.0%)		
Depression	Yes	19 (5.8)	10 (52.6%)	9 (47.4%)	χ^2^ = 0.020	0.888
	No	308 (94.2)	157 (51.0%)	151 (49.0%)		
Congestive heart failure	Yes	7 (2.1)	5 (71.4%)	2 (28.6%)	-	0.449^a^
	No	320 (97.9)	162 (50.6%)	158 (49.4%)		
Chronic kidney disease	Yes	33 (10.1)	24 (72.7%)	9 (27.3%)	χ^2^ = 6.889	0.009*
	No	294 (89.9)	143 (48.6%)	151 (51.4%)		
Chronic bronchitis / Chronic obstructive pulmonary disease (COPD)	Yes	39 (11.9)	20 (51.3%)	19 (48.7%)	χ^2^ = 0.001	0.978
	No	288 (88.1)	147 (51.0%)	141 (49.0%)		
Hyperlipidemia	Yes	92 (28.1)	36 (39.1%)	56 (60.9%)	χ^2^ = 7.303	0.007*
	No	235 (71.9)	131 (55.7%)	104 (44.3%)		
Number of comorbidities	1 type	191 (58.4)	139 (72.8%)	52 (27.2%)	χ^2^ = 86.780	<0.001*
	2 types	117 (35.8)	25 (21.4%)	92 (78.6%)		
	≥3 types	19 (5.8)	3 (15.8%)	16 (84.2%)		
Spouse’s HIV status	Positive	102 (31.2)	47 (46.1%)	55 (53.9%)	χ^2^ = 1.478	0.224
	Negative	225 (68.8)	120 (53.3%)	105 (46.7%)		
HIV knowledge learning experience	Yes	169 (51.7)	116 (68.6%)	53 (31.4%)	χ^2^ = 43.203	<0.001*
	No	158 (48.3)	51 (32.3%)	107 (67.7%)		
Albumin (g/L) Median (Q1, Q3)			43.08 (42.20, 44.10)	43.08 (42.45, 44.30)	Z = −0.156	0.876
Total cholesterol (mmol/L) Median (Q1, Q3)			4.65 (4.12, 4.72)	4.65 (4.33, 4.88)	Z = −0.815	0.415
Triglycerides (mmol/L) Median (Q1, Q3)			2.10 (1.30, 2.12)	2.10 (1.10, 2.10)	Z = −0.720	0.471
LDL-C (mmol/L) Median (Q1, Q3)			2.59 (2.28, 2.88)	2.59 (2.25, 2.71)	Z = −0.621	0.535
HDL-C (mmol/L) Median (Q1, Q3)			1.21 (1.03, 1.26)	1.21 (1.14, 1.27)	Z = −1.063	0.288
Glucose (mmol/L) Median (Q1, Q3)			6.37 (5.33, 6.37)	6.37 (5.33, 6.37)	Z = −0.067	0.947
Neutrophil count (×10^9^/L) Median (Q1, Q3)			3.55 (2.85, 3.72)	3.55 (2.83, 3.77)	Z = −0.323	0.747
Red blood cell count (×10^12^/L) Median (Q1, Q3)			4.21 (4.10, 4.56)	4.21 (4.11, 4.38)	Z = −1.138	0.255
White blood cell count (×10^9^/L) Median (Q1, Q3)			6.03 (5.21, 6.42)	6.03 (5.06, 6.22)	Z = −0.255	0.799
CD4/CD8 ratio Median (Q1, Q3)			0.83 (0.57, 0.86)	0.83 (0.58, 0.88)	Z = −0.600	0.549
Social support (SSRS score)	Low social support	36 (11.0)	1 (2.8%)	35 (97.2%)	χ^2^ = 41.688	<0.001^*^
	Moderate social support	266 (81.3)	147 (55.3%)	119 (44.7%)		
	High social support	25 (7.6)	19 (76.0%)	6 (24.0%)		
HIV/AIDS self-management scale Median (Q1, Q3)	Daily life management		12.00 (10.00, 14.50)	9.00 (6.00, 11.00)	Z = −8.747	<0.001^*^
	Disease knowledge		10.00 (8.00, 12.00)	7.00 (5.00, 9.00)	Z = −8.964	<0.001^*^
	Symptom management		26.00(24.00, 29.00)	21.00 (19.00, 25.50)	Z = −7.989	<0.001^*^
	Treatment adherence		24.00 (21.00, 28.00)	19.00 (16.00, 23.00)	Z = −7.564	<0.001^*^
	Emotional and cognitive management		19.00 (15.00, 21.00)	15.50 (13.00, 19.00)	Z = −5.271	<0.001^*^

*p < 0.05; SSRS, Social Support Rating Scale; MCI, Mild Cognitive Impairment; NC, Normal Cognition; Median (Q1, Q3), median (first quartile, third quartile); χ^2^, Chi-square test; Z, Mann–Whitney U test; ^a^, Fisher’s exact test.

### 3.2 Factors associated with mild cognitive impairment

#### 3.2.1 Univariate analysis

Univariate analysis was performed to explore factors associated with MCI among older adults living with HIV and multimorbidity. The results showed that there were statistically significant differences between the MCI group and the normal cognition (NC) group in terms of age, sex, residence, education level, employment status in the past 3 months, monthly income, type of medical expense payment, HIV transmission route, diabetes, hypertension, chronic kidney disease, hyperlipidemia, number of comorbidities, HIV knowledge learning experience, self-management ability, and social support (SSRS score; all *p* < 0.05). Detailed results are presented in Table 1.

#### 3.2.2 Logistic regression analysis

Binary logistic regression analysis was conducted with cognitive function as the dependent variable (1 = MCI, 0 = NC). Variables that were statistically significant in the univariate analysis were included as independent variables in the model. The assignment and coding of variables are shown in Table 2. The results indicated that education level (junior high school, High school and above), HIV knowledge learning experience, social support, and daily living management ability were protective factors for cognitive function (*p* < 0.05). In contrast, age (60–69 years and ≥70 years), female sex, monthly income (3,001–5,999 RMB), hypertension, and number of comorbidities were identified as significant risk factors for cognitive impairment (*p* < 0.05). Detailed results of the logistic regression analysis are shown in Table 3.

**Table 2:** Variable assignment and coding in binary logistic regression analysis.

Variable	Assignment and coding			
Dependent variable				
Cognitive function	NC = 0	MCI = 1		
Independent variables				
Age	50–59 years = 1	60–69 years = 2	≥70 years = 3	
Sex	Male = 1	Female = 2		
Residence	Urban = 1	Rural = 2		
Education level	Primary school or below = 1	Junior high school = 2	High school and above = 3	
Employment status in the past 3 months	Unemployed = 1	Occasional or short-term employment = 2	Stable long-term employment = 3	
Monthly income (RMB)	≤3,000 = 1	3,001–5,999 = 2	6,000–10,000 = 3	>10,000 = 4
Health insurance type	Self-paid = 1	Urban Resident Basic Medical Insurance = 2	New Rural Cooperative Medical Scheme = 3	
HIV transmission route	Men who have sex with men (MSM) = 1	Heterosexual transmission = 2	Other routes = 3	
Diabetes	No = 0	Yes = 1		
Hypertension	No = 0	Yes = 1		
Hyperlipidemia	No = 0	Yes = 1		
Chronic kidney disease	No = 0	Yes = 1		
Number of comorbidities	1 type = 1	2 types = 2	≥3 types = 3	
HIV knowledge learning experience	No = 0	Yes = 1		
SSRS	Low social support = 1	Moderate social support = 2	High social support = 3	
HIV/AIDS self-management scale	Daily life management	entered as original value		
	Disease knowledge	entered as original value		
	Symptom management	entered as original value		
	Treatment adherence	entered as original value		
	Emotional and cognitive management	entered as original value		

**Table 3:** Binary logistic regression analysis (*n* = 327).

Variable		*β*	SE	Wald χ^2^	*p* value	OR(95% CI)
Age	50–59 years (reference)					
	60–69 years	1.681	0.533	9.962	0.002	5.37 (1.891, 15.25)
	≥70 years	1.886	0.869	4.706	0.03	6.59 (1.2, 36.206)
Sex	Male (reference)					
	Female	1.37	0.62	4.879	0.027	3.934 (1.167, 13.261)
Residence	Urban (reference)					
	Rural	−0.667	0.617	1.169	0.28	0.513 (0.153, 1.72)
Education level	Primary school or below (reference)					
	Junior high school	−2.024	0.712	8.092	0.004	0.132 (0.033, 0.533)
	High school and above	−4.231	0.85	24.761	<0.001	0.015 (0.003, 0.077)
Employment status in the past 3 months	Unemployed (reference)					
	Occasional or short-term employment	−2.285	1.338	2.918	0.088	0.102 (0.007, 1.401)
	Stable long-term employment	−0.459	0.547	0.706	0.401	0.632 (0.216, 1.844)
Monthly income (RMB)	≤3,000 (reference)					
	3,001–5,999	1.326	0.574	5.346	0.021	3.767 (1.224, 11.594)
	6,000–10,000	1.118	0.893	1.566	0.211	3.058 (0.531, 17.613)
	>10,000	0.724	2.188	0.109	0.741	2.062 (0.028, 150.13)
Health insurance type	Self-paid (reference)					
	Urban Resident Basic Medical Insurance	0.637	1.659	0.147	0.701	1.89 (0.073, 48.844)
	New Rural Cooperative Medical Scheme	0.772	1.626	0.225	0.635	2.164 (0.089, 52.346)
HIV transmission route	Men who have sex with men (MSM) (reference)					
	Heterosexual transmission	−0.932	0.509	3.352	0.067	0.394 (0.145, 1.068)
	Other routes	−0.164	1.336	0.015	0.902	0.848 (0.062, 11.635)
Diabetes	No (reference)					
	Yes	1.133	0.604	3.518	0.061	3.104 (0.95, 10.137)
Hypertension	No (reference)					
	Yes	1.801	0.557	10.465	0.001	6.056 (2.034, 18.035)
Hyperlipidemia	No (reference)					
	Yes	0.549	0.617	0.794	0.373	1.732 (0.517, 5.801)
Chronic kidney disease	No (reference)					
	Yes	−1.189	0.809	2.16	0.142	0.305 (0.062, 1.487)
HIV knowledge learning experience	No (reference)					
	Yes	−1.202	0.508	5.587	0.018	0.301 (0.111, 0.814)
Number of comorbidities	1 (reference)					
	2	2.255	0.635	12.624	<0.001	9.533 (2.748, 33.065)
	≥3	3.106	1.239	6.281	0.012	22.337 (1.968, 253.528)
SSRS	Low social support (reference)					
	Moderate social support	−3.541	1.344	6.945	0.008	0.029 (0.002, 0.404)
	High social support	−4.341	1.603	7.33	0.007	0.013 (0.001, 0.302)
HIV/AIDS self-management scale	Daily life management	−0.202	0.097	4.301	0.038	0.817 (0.675, 0.989)
	Disease knowledge	−0.14	0.1	1.94	0.164	0.87 (0.714, 1.059)
	Symptom management	−0.001	0.053	0.001	0.981	0.999 (0.9, 1.109)
	Treatment adherence	0.026	0.051	0.263	0.608	1.027 (0.928, 1.136)
	Emotional and cognitive management	−0.05	0.062	0.65	0.42	0.951 (0.841, 1.075)

#### 3.2.3 Decision tree model analysis

A decision tree model was constructed with cognitive function as the dependent variable and variables that were statistically significant in the univariate analysis (*p* < 0.05) as independent variables. The results showed that the constructed decision tree model had a depth of three levels, including 15 nodes and 9 terminal nodes. A total of five key predictors were identified: number of comorbidities, education level, age, hypertension and HIV knowledge learning experience.

**Figure 1:**
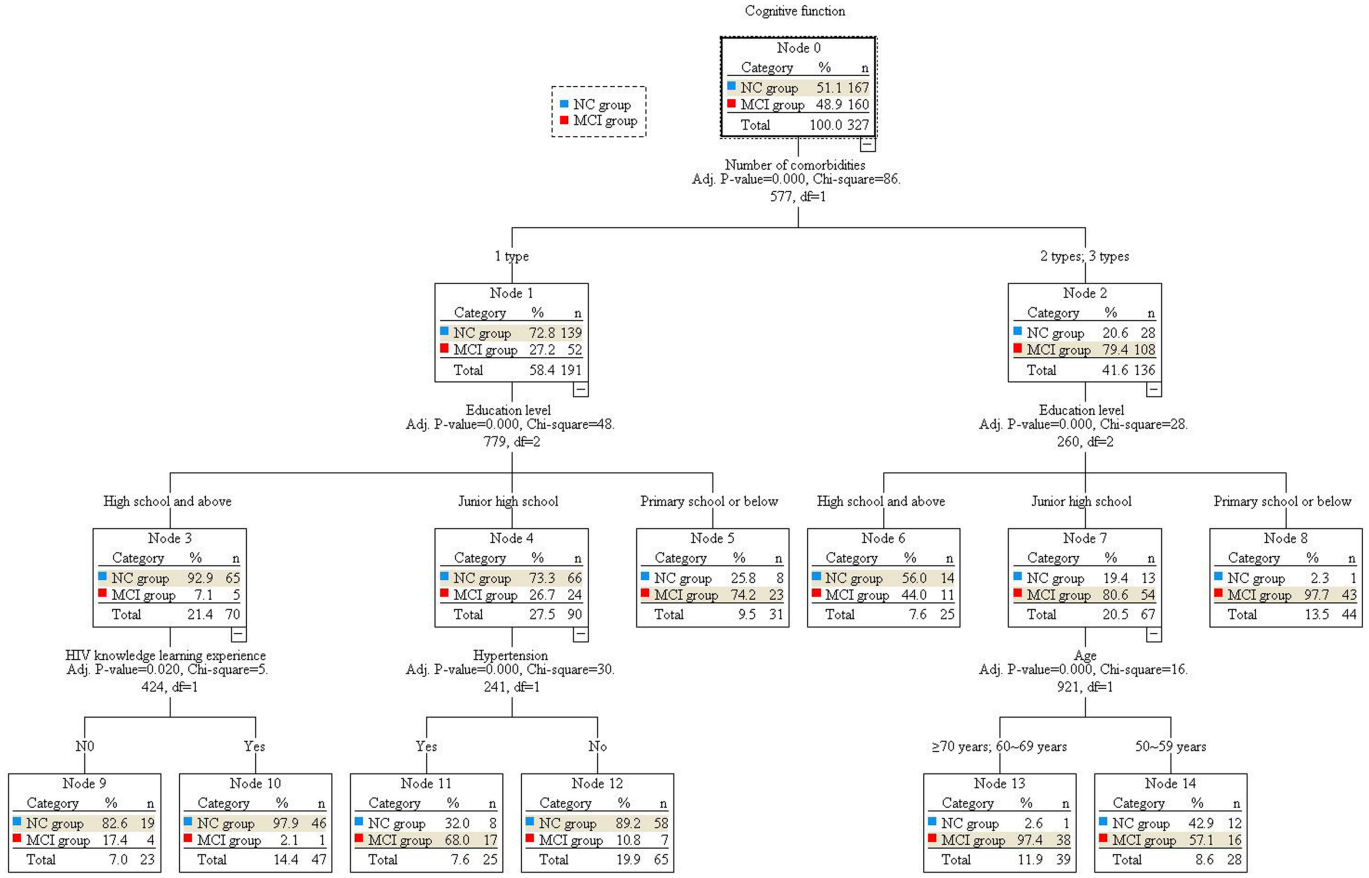
CHAID decision tree model for screening-defined mild cognitive impairment among older adults living with HIV and multimorbidity.

The first level of the tree structure was number of comorbidities, indicating that it was the most important predictor of MCI among older adults living with HIV. The detection rate of MCI among participants with two or three comorbidities (79.4%) was significantly higher than that among those with only one comorbid condition (27.2%). At the second level, education level further stratified the risk. Participants with primary school education or below showed higher detection rates of MCI in different comorbidity subgroups (97.7 and 74.2%, respectively). At the third level, age, hypertension, and HIV knowledge learning experience were identified as additional important variables. Participants with a higher number of comorbidities, junior high school education level, and older age had the highest detection rate of MCI (97.4%). Among participants with only one comorbid condition and a junior high school education level, those with hypertension had a significantly higher prevalence of MCI compared with those without hypertension (68.0% vs. 10.8%). In contrast, participants with higher education levels and prior HIV knowledge learning experience had relatively lower rates of MCI (2.1% vs. 17.4%). The structure of the decision tree model is shown in Figure 1.

#### 3.2.4 Comparison between logistic regression and decision tree models

**Figure 2:**
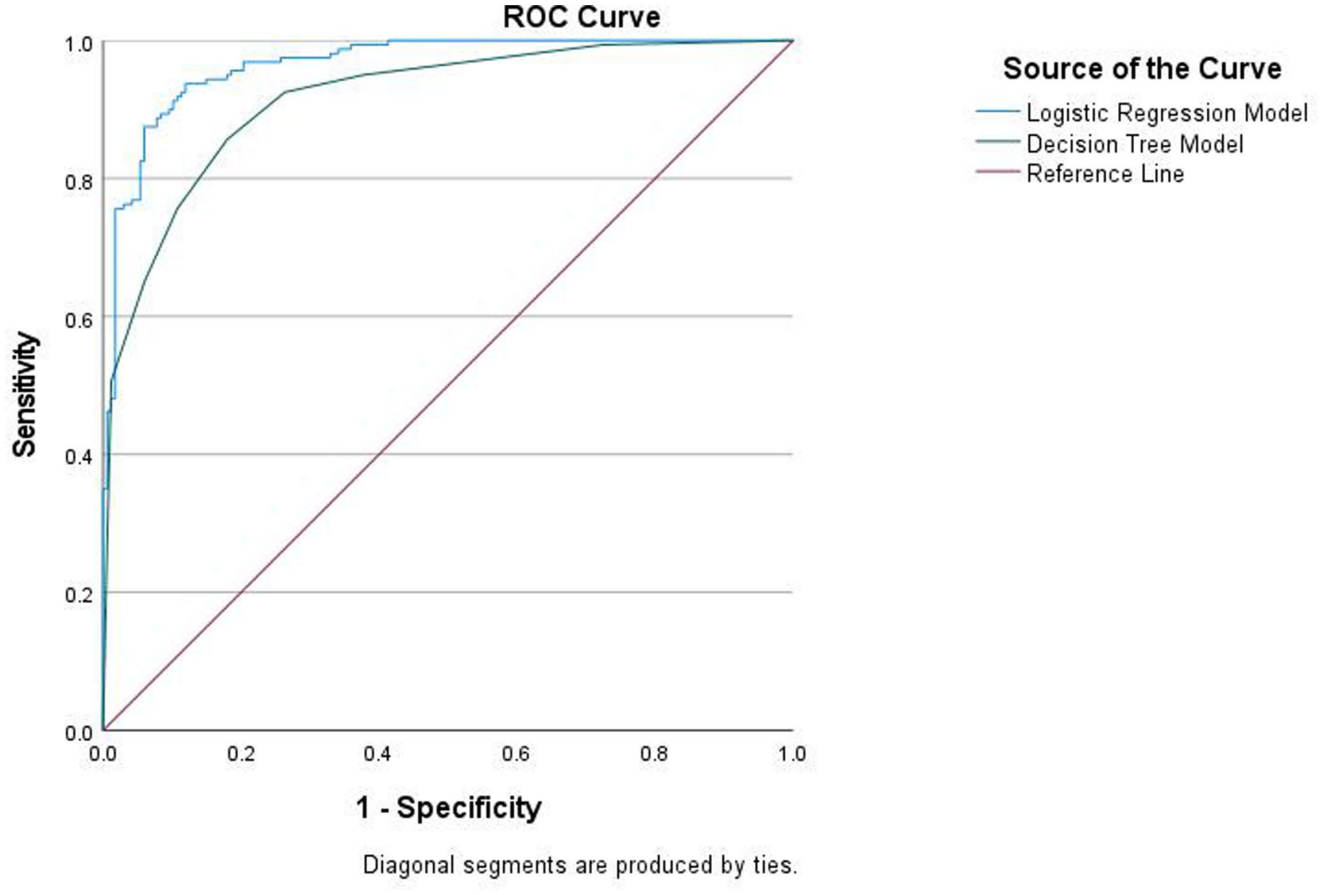
Receiver operating characteristic (ROC) curves comparing the predictive performance of the logistic regression model and the decision tree model.

The sensitivity, specificity, and Youden index of the logistic regression model were slightly higher than those of the decision tree model. The area under the ROC curve (AUC) for the logistic regression model was 0.961 (95% CI: 0.942–0.979), while the AUC for the decision tree model was 0.916 (95% CI: 0.886–0.946). Both models showed statistically significant classification performance (*p* < 0.001). The difference between the AUCs of the two models was statistically significant (Z = 3.935, *p* < 0.01), indicating that the logistic regression model had superior predictive performance compared with the decision tree model. Detailed results are presented in Table 4 and Figure 2.

**Table 4:** Comparison of predictive performance between the logistic regression model and the decision tree model.

Model	AUC	Standard error	95%CI	*p* value	Sensitivity	Specificity	Youden index
Logistic regression model	0.961	0.009	0.942–0.979	<0.001	0.894	0.922	0.816
Decision tree model	0.916	0.015	0.886–0.946	<0.001	0.856	0.820	0.676

## 4 Discussion

This study investigated the prevalence and factors associated with MCI among older adults living with HIV and multimorbidity, and applied both logistic regression and decision tree approaches to explore factors associated with MCI and their potential predictive value. The prevalence of screening-defined MCI in this population was 48.9%, which is higher than that reported in community-dwelling older adults in the general population (20). This finding suggests that cognitive impairment represents an important health concern among older adults living with HIV and comorbid conditions. It is possible that age-related neurodegenerative processes may coexist with HIV-associated neurocognitive injury (21), although such mechanisms cannot be established in the present cross-sectional study.

All participants in this study had at least one chronic comorbid condition. Both logistic regression and decision tree models consistently identified the number of comorbidities as a key factor associated with MCI, and it was the primary splitting variable in the decision tree model. A greater burden of chronic diseases has been linked in previous studies to systemic inflammation and metabolic dysregulation, which are associated with cognitive impairment (22). In addition, the coexistence of multiple chronic conditions may increase treatment complexity and medication burden, potentially influencing disease management and quality of life (23). These findings underscore the importance of comprehensive management of multimorbidity in this population.

Education level was identified as a protective factor against MCI, which is consistent with previous studies (24). According to the cognitive reserve theory, higher educational attainment is associated with greater cognitive reserve and may help individuals better cope with neuropathological changes (25). Individuals with higher education levels may also have better health literacy and access to healthcare resources, which may be associated with more favorable cognitive outcomes.

Age and hypertension were also identified as important risk factors for cognitive decline. With advancing age, structural and functional changes in the central nervous system, including reduced cerebral blood flow and neuronal degeneration, have been reported in prior studies (26). Hypertension may further accelerate cognitive decline by damaging cerebral vasculature, impairing cerebral blood supply, and promoting atherosclerosis (27). Therefore, regular blood pressure monitoring and effective management of chronic conditions should be emphasized in the care of older adults living with HIV.

Interestingly, traditional HIV-related clinical indicators, including CD4 + T-cell count, HIV viral load, and CD4/CD8 ratio, were not significantly associated with MCI in this study. One possible explanation is that most participants were receiving long-term antiretroviral therapy (ART), with 85.0% treated for more than 18 months and 88.1% achieving virologic suppression. This relatively homogeneous clinical profile may have reduced variability in these indicators, thereby limiting the ability to detect potential associations. In the context of effective ART, it has been suggested that cognitive impairment among older adults living with HIV may be increasingly associated with aging, multimorbidity, and other non-HIV-related factors rather than traditional HIV disease markers (28). This may help explain why variables such as number of comorbidities, age, and hypertension emerged as more prominent correlates in the present study.

This study also found that female participants had a higher risk of MCI compared with males, which is consistent with previous findings (29). This may be explained by biological differences, such as hormonal changes and pharmacokinetic variations, as well as social determinants, including lower socioeconomic status, poorer mental health, and reduced access to healthcare services.

With regard to socioeconomic factors, participants with a monthly income of 3,001–5,999 RMB had a higher likelihood of MCI compared with those with lower income (≤3,000 RMB). This finding is somewhat unexpected and should be interpreted with caution. One possible explanation is that income level may be correlated with other factors in this sample, such as health insurance type and urban–rural residence, which may partially confound the observed association. In addition, income may act as a proxy for other unmeasured variables, including financial burden, access to healthcare, and psychosocial stress. Although differences in social welfare and medical assistance policies across income groups may play a role, this interpretation remains speculative and is not directly supported by the data collected in this study. Further research is needed to better understand this relationship.

Furthermore, this study demonstrated that HIV knowledge learning experience and social support were protective factors for cognitive function. Participants who had received HIV-related education had a lower prevalence of MCI, possibly because health education improves disease awareness, promotes treatment adherence, and encourages healthy behaviors. Social support from family and society may help alleviate stress, enhance coping capacity, and improve disease management, thereby contributing to the maintenance of cognitive function. These findings highlight the importance of strengthening health education and social support interventions for older adults living with HIV.

In terms of analytical approaches, both logistic regression and the CHAID decision tree were applied to examine factors associated with MCI and to explore their potential discriminative ability. The logistic regression model demonstrated a higher apparent AUC compared with the decision tree model; however, this performance was evaluated using the same dataset on which the model was developed and therefore may be subject to overestimation. In contrast, the decision tree model incorporated 10-fold cross-validation, providing a degree of internal validation and offering an interpretable structure that illustrates hierarchical relationships among predictors.

Importantly, both analytical approaches consistently identified key factors—such as number of comorbidities, age, hypertension, and education level—highlighting the robustness of these associations across different methods. These findings suggest that the observed relationships are unlikely to be solely model-dependent. Overall, the results of this study should be considered exploratory. While the models provide useful insights into potential risk stratification, further studies incorporating rigorous internal and external validation are needed to confirm their predictive performance and generalizability.

## 5 Limitations

Several limitations should be acknowledged in this study. First, the cross-sectional design precludes the establishment of causal relationships between the identified factors and cognitive impairment. Longitudinal studies are needed to further clarify these associations.

Second, participants were recruited from a single center, which may introduce selection bias and limit the generalizability of the findings. Future multicenter studies with larger sample sizes are warranted to validate these results.

Third, some variables were collected through self-report, which may be subject to recall bias. In addition, cognitive impairment was identified using screening instruments rather than comprehensive neuropsychological and functional assessments, which may have limited the precision of cognitive classification.

Finally, although several HIV-related clinical indicators (e.g., CD4 + T-cell count, viral load, and CD4/CD8 ratio) were included, other important variables, such as specific antiretroviral treatment regimens and CD4 percentage, were not collected. Moreover, the relatively well-controlled HIV status of most participants may have reduced variability in these indicators, limiting the ability to detect potential associations.

Future research should focus on multicenter, large-scale longitudinal studies to further validate these findings and develop more precise predictive models for early identification and intervention of cognitive impairment among older adults living with HIV.

## 6 Conclusion

Mild cognitive impairment is highly prevalent among older adults living with HIV and multimorbidity. Multiple factors—including number of comorbidities, age, hypertension, education level, socioeconomic characteristics, and behavioral factors—were found to be associated with MCI. The application of both logistic regression and decision tree approaches yielded broadly consistent findings, suggesting that these factors may represent robust correlates of cognitive impairment in this population. However, the results of this study should be interpreted as exploratory, given the cross-sectional design and limited model validation. Further longitudinal studies with larger and more diverse samples, as well as rigorous internal and external validation, are needed to confirm these findings and to support the development of reliable risk prediction tools.

## Data Availability

The original contributions presented in the study are included in the article/supplementary material, further inquiries can be directed to the corresponding author.
